# Spectral reflectance data of a high temperature stable solar selective coating based on MoSi_2_*–*Si_3_N_4_

**DOI:** 10.1016/j.dib.2016.04.040

**Published:** 2016-04-23

**Authors:** D. Hernández-Pinilla, A. Rodríguez-Palomo, L. Álvarez-Fraga, E. Céspedes, J.E. Prieto, A. Muñoz-Martín, C. Prieto

**Affiliations:** a Instituto de Ciencia de Materiales de Madrid, Consejo Superior de Investigaciones Científicas, Cantoblanco, 28049 Madrid, Spain; b Dpto. Física de Materiales, Universidad Autónoma de Madrid, E-28049 Madrid, Spain; c IMDEA Nanociencia, E-28049 Madrid, Spain; d Centro de Microanálisis de Materiales, Universidad Autónoma de Madrid, 28049 Madrid, Spain; e Dpto. Física de la Materia Condensada, Universidad Autónoma de Madrid, 28049 Madrid, Spain

**Keywords:** Solar selective coating

## Abstract

Data of optical performance, thermal stability and ageing are given for solar selective coatings (SSC) based on a novel MoSi_2_–Si_3_N_4_ absorbing composite. SSC have been prepared as multilayer stacks formed by silver as metallic infrared reflector, a double layer composite and an antireflective layer (doi: 10.1016/j.solmat.2016.04.001 [1]). Spectroscopic reflectance data corresponding to the optical performance of samples after moderate vacuum annealing at temperatures up to 600 °C and after ageing test of more than 200 h with several heating–cooling cycles are shown here.

## 1 Specifications Table

**Table:** 

Subject area	Materials
More specific subject area	Optical properties
Type of data	Figure
How data was acquired	Shimadzu SolidSpec-3700 spectrophotometer (wavelength range: 190–2600 nm) and Varian 660-IR FTIR spectrometer (wavelength range: 1.5–25 µm)
Data format	Analyzed
Experimental factors	As-deposited and vacuum annealed post-deposition treatment of solar selective films onto stainless steel substrates
Experimental features	BaSO_4_ and Au used as standards for 100% reflectance in visible and infrared wavelengths, respectively.
Data source location	Madrid, Spain
Data accessibility	With this article

## 2 Value of the data

• The reported data allow direct comparison with other reflectance spectra of solar selective coatings (SSC) based on different absorbers and/or layers characteristics. Data provides the fundamental characterization of the optical properties of SSC used in concentrated solar power (CSP) systems.
• The reflectance data are valuable for a full optical and thermal analysis of the potential of the SSC in a solar field of a parabolic trough concentrator system. For instance by using a 3D heat-transfer model [1], [2], reflectance data let calculate the optical efficiency of the whole concentrated solar power (CSP) system and therefore the heat gain and overall thermal efficiency.
• The spectral reflectance data are valuable for the optimization of improving selectivity (i.e. its solar absorptivity/thermal emissivity) in solar concentrating systems as a function of the operation temperature.
• Data after long annealing periods at high temperature allows straight evaluation and comparison of the thermal stability and durability of this coating versus other investigated SSC.

## 3 Data

Spectral reflectance of a representative solar selective coating based on MoSi_2_–Si_3_N_4_ cermet measured after 50, 150 and 200 h of vacuum annealing at 600 °C. The wavelength measured (200–30,000 nm) is the whole UV–vis–IR range of interest for CSP applications, covering solar irradiance and the black body emission at operating temperatures.

## 4 Experimental design, materials and methods

The solar selective tandems were deposited by magnetron sputtering at room temperature on stainless steel AISI-321 substrates (with a previous air-annealing at 600 °C to develop its thermally grown oxide anti-diffusion layer). The stack materials were: silver as metallic IR reflector, MoSi_2_/Si_3_N_4_ composites as high metal volume fraction (HMVF) and low metal volume fraction (LMVF) absorber composite layers and silicon nitride as antireflective layer on top. The description of coating preparation is given in the experimental section of the associated research article [1].

**Fig. 1:**
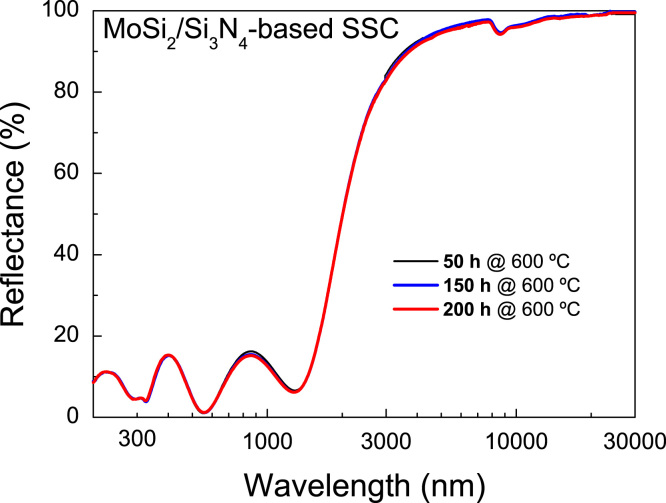
Reflectance spectra of a representative selective coating after long time periods (50, 150 and 200 h) of vacuum annealing at 600 °C.

The here reported coating is comprised of Ag (100 nm)/HMVF (FF=50%, 75 nm)/LMVF (FF=15%, 55 nm)/Si_3_N_4_ (53 nm). Fig. 1 shows the reflectance spectra obtained after several cycles of vacuum annealing at 600 °C. Data are also reported in Fig. 1. By using the well-known expressions (given for instance in Ref. [3]) for the solar absorptivity (*α*_*Sol*_) and thermal emissivity (*ε*_*th*_(*T*)), the performance of this solar selective coating is characterized by *α*_*Sol*_=88% and *ε*_*th*_(25 °C)=2%, *ε*_*th*_(450 °C)=8%, *ε*_*th*_(600 °C)=11%. Those values allow selectivity ratios of *ξ*_(25_ _°C)_=44, *ξ*_(450__°C)_=11 and *ξ*_(600__°C)_=8; with *ξ*_(*T*)_ defined as *ξ*_(*T*)_=*α*_*Sol*_/*ε*_*th*_(*T*).
