# Supplementary material for: Spectral reflectance data of a high temperature stable solar selective coating based on MoSi2–Si3N4
Source: Data Brief. 2016 Apr 23;7:1483–5. doi: 10.1016/j.dib.2016.04.040 (PMC4857402; doi:10.1016/j.dib.2016.04.040)
Supplement: Supplementary file 1 — Supplementary material [file mmc1.pdf]

MINISTERIO
DE ECONOMÍA
Y COMPETITIVIDAD

CSIC
CONSEJO SUPERIOR DE INVESTIGACIONES CIENTÍFICAS
INSTITUTO DE CIENCIA DE MATERIALES
DE MADRID (ICMM)

Sarah O'Loughlin
Managing Editor
Data in Brief

Dear Editor,

All co-authors of this manuscript declare to have not conflicts of interest.

Sincerely

Prof. Carlos Prieto
Instituto de Ciencia de Materiales de Madrid,
Consejo Superior de Investigaciones Científicas

SOR JUANA INÉS DE LA CRUZ, 3
CANTOBLANCO
28049 MADRID. ESPAÑA
TEL.: 91 334 90 00
FAX: 91 372 06 23
http://www.icmm.csic.es
